# The Effect of Narrow-Band Ultraviolet B Phototherapy on Free and Total Vitamin D Serum Levels in Mild to Severe Plaque Psoriasis

**DOI:** 10.3390/biom13071018

**Published:** 2023-06-21

**Authors:** Andrea Elmelid, Maria Siekkeri Vandikas, Martin Gillstedt, Amra Osmancevic, Mikael Alsterholm

**Affiliations:** 1 Department of Dermatology and Venereology, Institute of Clinical Sciences, Sahlgrenska Academy, University of Gothenburg, 413 45 Gothenburg, Swedenamra.osmancevic@vgregion.se (A.O.); mikael.alsterholm@vgregion.se (M.A.); 2 Department of Dermatology and Venereology, Falu Hospital, Region Dalarna, 791 82 Falun, Sweden; 3 Dermatology and Venereology Unit, Karolinska University Hospital, 171 76 Stockholm, Sweden; 4 Department of Dermatology and Venereology, Sahlgrenska University Hospital, Region Västra Götaland, 413 45 Gothenburg, Sweden; 5 Dermatology and Venereology Unit, Department of Medicine Solna, Karolinska Institutet, 171 76 Stockholm, Sweden

**Keywords:** vitamin D, free 25-hydroxy vitamin D, psoriasis, phototherapy, ultraviolet B therapy, inflammation

## Abstract

Vitamin D plays an important role in skin inflammation in psoriasis. The beneficial effects of ultraviolet light B (UVB) phototherapy in psoriasis are partly attributed to UVB-induced increase of vitamin D levels. In clinical practice, total 25-hydroxy vitamin D (25(OH)D) levels are measured to assess sufficiency, but it might be more accurate to measure free 25(OH)D levels. The aim of this study was to measure free serum 25(OH)D levels in psoriasis patients before and after phototherapy and to investigate if free 25(OH)D correlates stronger to disease severity than total 25(OH)D. Twenty adults (>18 years) with psoriasis were included for treatment with narrow-band UVB (NB-UVB) phototherapy for 10–12 weeks. Psoriasis Area and Severity Index (PASI) and Visual Analogue Scale (VAS) were used to assess disease severity. Serum levels of total 25(OH)D, free 25(OH)D, and 1,25(OH)_2_D were measured before and after NB-UVB. Total 25(OH)D, free 25(OH)D, 1,25(OH)_2_D and the percentage of free 25(OH)D increased after NB-UVB, and PASI and VAS improved. The increase in total and free 25(OH)D remained significant when stratifying for vitamin D confounders. No correlations between disease severity and vitamin D levels were found. Total and free 25(OH)D levels were positively correlated before and after NB-UVB. NB-UVB is an effective treatment for mild to severe plaque psoriasis and increases not only total but also free 25(OH)D levels, as well as the percentage of free 25(OH)D, suggesting an increased bioavailability of skin-produced vitamin D.

## 1. Introduction

Psoriasis is a chronic, immune-mediated inflammatory skin disease that affects approximately 2–3% of the population worldwide. Patients with psoriasis suffer from substantial morbidity and increased risk of cardiometabolic diseases, mental health disorders, and psoriatic arthritis [1].

It is established that vitamin D plays a role in psoriasis but whether it is involved in its pathogenesis remains unclear [2,3]. Topically applied vitamin D is an effective and established first-line treatment for plaque-type psoriasis. However, studies have yielded inconsistent results regarding the benefits of oral vitamin D supplements [4].

One possible explanation for the conflicting results could be the use of an inaccurate biomarker for the assessment of vitamin D status in psoriasis patients.

According to the free hormone hypothesis, the action of a steroid hormone is mediated through the unbound fraction whereas the protein-bound hormone is biologically inactive. For steroid hormones such as thyroid hormones and sex hormones, the free concentration is measured in clinical practice [5]. In contrast, vitamin D status is determined by measuring total 25-hydroxy D (25(OH)D) levels. Less than 1% of total serum 25(OH)D is circulating in its free form, while the major fraction is tightly bound to vitamin D binding protein (DBP) or loosely bound to albumin.

DBP is the major transport protein for all vitamin D metabolites. The DBP concentration and the protein’s affinity for 25(OH)D define the amount of free 25(OH)D. In conditions such as pregnancy and liver or kidney disease, DBP levels are altered, resulting in a shift of the equilibrium between total and free 25(OH)D [5,6].

Previously, a mathematical model has been used to calculate the free 25(OH)D level. Since the arrival of a two-step enzyme-linked immunosorbent assay (ELISA) that measures free 25(OH)D directly, studies have shown that the calculated values of free 25(OH)D highly overestimate the measured levels of free 25(OH)D [5]. Directly measured free 25(OH)D has previously been studied in psoriasis patients, but there are no studies on the effect of NB-UVB phototherapy on free 25(OH)D levels in this group [7,8,9].

In recent years, the extra-skeletal impact of vitamin D, especially the effects on the immune system, has received much attention. When human skin is irradiated with ultraviolet B light (UVB) (290–315 nm) pre-vitamin D_3_ is produced from 7-dehydrocholesterol (7-DHC). Although vitamin D can be obtained from the diet, including fortified food items, skin-produced vitamin D constitutes the main source for the body [10]. Vitamin D is converted to its most active form 1,25-dihydroxy D (1,25(OH)_2_D), by 1-alpha-hydroxylase [11]. Initially thought to be specific to the kidney, 1-alpha-hydroxylation has later been found to occur in extra-renal cells such as keratinocytes and immune cells [12,13]. The function of 1,25(OH)_2_D is mediated by the nuclear vitamin D receptor (VDR). VDR is expressed by keratinocytes making the skin both a source and a target for vitamin D [14].

Phototherapy is widely used in the treatment of psoriasis. Several studies have reported a positive effect of UVB phototherapy on serum 25(OH)D levels in psoriasis patients [15,16]. The beneficial effects are partly attributed to the increase in vitamin D levels that occurs after the skin is exposed to UVB [2].

The aim of this study was to measure serum levels of free and total 25(OH)D in psoriasis patients before and after narrow-band UVB (NB-UVB) phototherapy. We hypothesized that phototherapy would increase both total and free 25(OH)D levels and that free 25(OH)D levels would correlate stronger to disease severity than total 25(OH)D.

## 2. Materials and Methods

### 2.1. Study Design, Setting, and Participants

Twenty adult (>18 years) patients with mild to severe plaque psoriasis (determined by Psoriasis Area and Severity Index (PASI)) were recruited from the outpatient clinic at the Department of Dermatology and Venereology at Sahlgrenska University Hospital, Gothenburg, Sweden, between 2013–2017.

Multiple exclusion criteria relevant to vitamin D status were applied, as previously described [17].

Patients receiving their first treatment (visit 1) in October to March, when vitamin D production in the skin is negligible due to lack of natural UVB exposure, were defined as winter treated. Those whose first visit took place in April to September were defined as summer treated. From April to September, the UV index in Gothenburg can reach ≥3 and vitamin D synthesis in the skin can occur.

Before treatment, skin type according to Fitzpatrick was determined [18]. Investigator- and patient-reported outcome measures, blood samples, weight, height, and blood pressure were collected at up to three-time points (visits 1–3) as previously described [17]. Questionnaires about vitamin D-confounders were obtained at every visit. PASI evaluation was made by a dermatologist at visits 1 and 3 (when treatment was ended at weeks 10–12).

According to the Endocrine Society, vitamin D sufficiency was defined as total 25(OH)D serum levels ≥ 75 nmol/L. Insufficiency was defined as 25(OH)D 50–74 nmol/L, and deficiency was defined as 25(OH)D < 50 nmol/L [19].

### 2.2. Phototherapy

All patients received treatment with NB-UVB (311–312 nm), Corona 4, ESSHÅ, two to three times per week for 10–12 weeks (maximum number of treatments 30). The UV doses were individually adjusted, according to a standardized protocol. As part of the standard procedure, the patients were permitted to use emollients and mild topical corticosteroids as needed. Topical calcineurin inhibitors and potent topical corticosteroids were not allowed.

### 2.3. Laboratory Analyses and Calculation of Percentage of Free 25(OH)D

Blood samples were analyzed as unicates at the Department of Clinical Chemistry, Sahlgrenska University Hospital, Gothenburg, Sweden.

To analyze total 25(OH)D [25(OH)D_2_ and 25(OH)D_3_] serum levels, an Electrochemiluminescence immunoassay (ECLIA) (Elecsys Vitamin D Total II assay) was used. The coefficient of variance (CV) was 12% at 66 nmol/L and 17% at 26 nmol/L. The limit of quantification (LoQ) was <10 nmol/L and >500 nmol/L.

A two-step immunosorbent assay (ELISA) (Future Diagnostics B.V., Wijchen, The Netherlands) was used to measure the free 25(OH)D serum levels. The CV was 9% at 4 ng/L. The LoQ was <1.8 ng/L and >40 ng/L.

An automated chemiluminescence immunoassay (CLIA) was used to analyze serum 1,25(OH)_2_D. The CV was 10% at 80 pmol/L and 15% at 130 pmol/L. The LoQ was <18 pmol/L.

Serum intact parathyroid hormone (iPTH) was analyzed with ECLIA with an Elecsys PTH STAT (Roche Diagnostics Scandinavia AB, Tokyo, Japan). The CV for iPTH was 7% at the level of 3 pmol/L and 3% at 10 pmol/L. The LoQ was <0.5 pmol/L and >530 pmol/L.

A detailed description of the analyses, and the method for the calculation of the percentage of free 25(OH)D, are reported earlier [20].

### 2.4. Assessment of Psoriasis Severity

Psoriasis Area and Severity Index (PASI) was used as the investigator-reported outcome measure for disease severity. PASI evaluates the involved body surface area and the appearance of lesions (erythema, induration, and scaling) with a range from 0–72 points, where 0 indicates no disease and 72 maximal diseases. A PASI score ≥ 10 indicates severe disease.

The patient-reported outcome measure (PROM) for psoriasis severity was the Visual Analogue Scale (VAS), where 0 corresponds to no psoriasis-related distress, and 10 corresponds to maximal psoriasis-related distress.

### 2.5. Statistics

Data are presented as mean ± SD, mean (min–max) or median (inter-quartile range (IQR)) if not otherwise stated. Data were analyzed using R version 3.5.3 (The R Foundation for Statistical Computing, Vienna, Austria). Simple descriptive statistics were applied. Spearman’s correlation test was used to test univariate correlations. Wilcoxon’s rank sum test was used for two sample tests. Spearman’s correlation test stratifying with respect to the patient was used when testing for changes over time. Wilcoxon’s signed rank test was used to test differences in PASI and biochemical data before and after NB-UVB. All tests were two-sided and *p* < 0.05 was considered statistically significant.

### 2.6. Ethical Considerations

The study was approved by the Ethics Committee at the University of Gothenburg on 22 May 2012 (approval number: 089-12). Declarations of Helsinki protocols were followed. Written informed consent was obtained from all patients.

## 3. Results

### 3.1. Demographics

Twenty patients with psoriasis were included. Fifteen patients completed the study. One patient was excluded from the study after the first visit due to a planned sun vacation. The remaining four patients lost to follow-up did not state a reason. Baseline data were analyzed for all twenty included patients.

The mean age of the patients was 44.3 ± 15.7 years and the mean disease duration was 19.5 ± 13.5 years.

Pretreatment PASI at visit 1 ranged from 3.9–21.0, which corresponds to mild to severe disease. Median PASI was 8.4 (7.6–12.2). Eighteen (90%) patients started NB-UVB phototherapy (visit 1) during winter, as defined in Section 2.

Most subjects had normal blood pressure and were of normal weight. A BMI of >30 kg/m^2^ was classified as obesity. Eleven (55%) patients reported arthropathy. Eleven (55%) patients reported psoriasis heredity. Demographic data and possible confounders for vitamin D status are presented in Table 1.

### 3.2. Effect of NB-UVB Treatment

The mean number of treatments (patients lost to follow-up excluded) was 26 ± 2.7 and the mean cumulative UVB dose was 36.0 ± 16.2 J/cm^2^.

Median PASI decreased from 8.4 (7.6–12.2) to 2.6 (1.2–3.2) (*p* = 0.0001). The mean PASI change was −7.1 ± 4.5. Median VAS decreased from 7.7 (7.0–8.7) to 2.1 (0.9–3.0) (*p* = 0.002).

**Figure 1:**
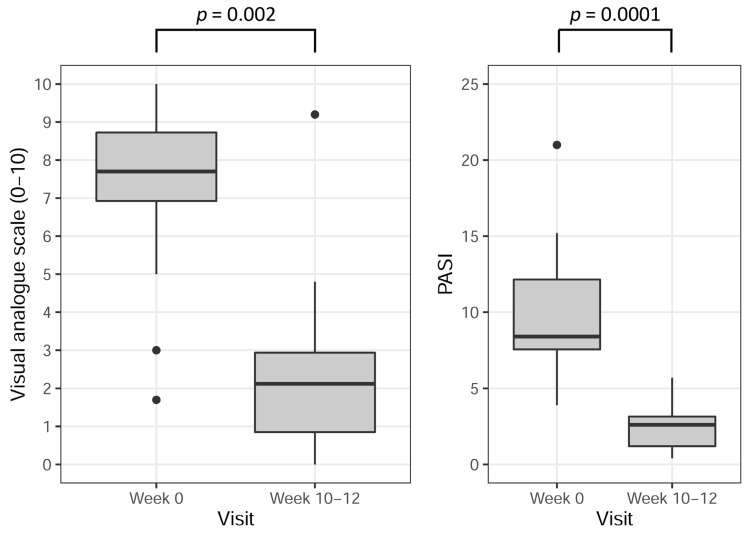
Visual Analogue Scale (VAS) and Psoriasis Area and Severity Index (PASI) before (visit 1 = week 0), and after (when treatment was ended at weeks 10–12 = visit 3) narrow-band ultraviolet light B (NB-UVB) phototherapy in psoriasis patients. The black dots are considered outliers by the boxplot function.

The effect of NB-UVB treatment on PASI and VAS are presented in Figure 1.

Total 25(OH)D, free 25(OH)D and 1,25(OH)_2_D levels increased significantly from visit 1 (before therapy) to visit 3 (when treatment was ended). Mean delta 25(OH)D was 50 ± 17 nmol/L and the mean delta free 25(OH)D was 11 ± 4.5 pmol/L. The percentage increase of total 25(OH)D was 95%, free 25(OH)D increased by 124%, 1,25(OH)_2_D increased by 13%, and the percentage of free 25(OH)D increased by 9.2%, as shown in Table 2.

Body mass index (BMI) did not change during NB-UVB treatment. Systolic blood pressure decreased significantly (*p* = 0.0004), but diastolic blood pressure varied with no clear trend (*p* = 0.61). iPTH decreased from 3.5 ± 0.7 pmol/L to 2.8 ± 0.6 pmol/L (*p* = 0.0012) from visit 1 to visit 3.

### 3.3. Vitamin D Status and Correlation to Disease Severity

At baseline, six (30%) patients had sufficient levels of 25(OH)D, eight (40%) had insufficient levels, and six (30%) were vitamin D deficient.

**Figure 2:**
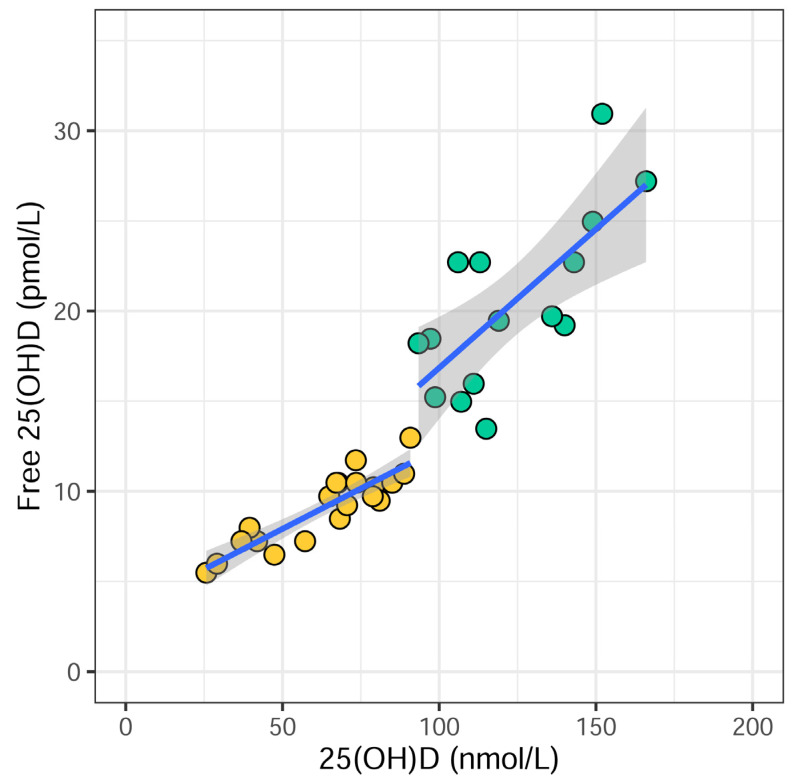
The correlation between serum levels of free 25(OH)D and total 25(OH)D before (yellow, *n* = 20) and after (when treatment was ended) (green, *n* = 15) narrow-band ultraviolet light B (NB-UVB) phototherapy in psoriasis patients. *p* < 0.0001 and *p* < 0.003, respectively. The blue lines are fitted linear regression lines and the grey areas are 95% confidence intervals for the fitted lines.

Free and total 25(OH)D concentrations were positively correlated at baseline (*p* < 0.0001) and after NB-UVB (*p* < 0.003), as shown in Figure 2.

No correlations were found between disease severity (as determined by PASI and VAS) and levels of any vitamin D metabolite including the percentage of free 25(OH)D at baseline or after NB-UVB. VAS and PASI did not correlate at baseline or after NB-UVB.

## 4. Discussion

To the best of our knowledge, this is the first report on the effects of phototherapy on free 25(OH)D serum levels in psoriasis patients. NB-UVB phototherapy raised serum levels of total 25(OH)D, free 25(OH)D, and 1,25(OH)_2_D. The percentage increase of total 25(OH)D levels after NB-UVB phototherapy in this cohort was 95%. This is in accordance with previous reports on psoriasis patients after NB-UVB (59–250% increase) [21]. The increase of 1,25(OH)_2_D, which is the active vitamin D metabolite, was much lower than the increase of 25(OH)D. This is in line with the strict endocrine regulation of the production of the active metabolite to avoid hypercalcemia. Previous studies have reported unaltered levels of 1,25(OH)_2_D after NB-UVB. However, one study on patients with insufficient levels of 25(OH)D (<75 nmol/L) before phototherapy showed a significant increase of 1,25(OH)_2_D post-treatment [22,23]. According to the sub-analysis of our data, the effects of NB-UVB on total and free 25(OH)D levels remained significant when stratifying for possible confounding factors for vitamin D, except for patients with BMI ≥ 30 kg/m^2^. However, this was probably due to the small sample size, Table 3.

As expected, NB-UVB was an effective treatment for psoriasis and demonstrated a significant improvement in PROMs and investigator-reported outcome measures. Future studies are needed to evaluate the long-term effects on vitamin D status and disease severity after cessation of phototherapy.

Interestingly, NB-UVB also increased the percentage of free 25(OH)D in psoriasis patients. This is a new insight into the biological effects of phototherapy on skin-produced vitamin D, showing that NB-UVB raises the bioavailable vitamin D levels.

To the best of our knowledge, it is not known whether oral substitution with vitamin D_3_, which is the standard treatment for vitamin D insufficiency, has similar effects on the percentage of free 25(OH)D. In a previous study in mice, Duchow et al. reported that skin produced 25(OH)D had a twofold higher bioactivity (measured by an increase in serum calcium) compared to oral vitamin D_3_ [24]. An increase in the percentage of free 25(OH)D might play a role in the increased bioactivity described.

Possibly, NB-UVB causes alterations in binding proteins (DBP and albumin), either by decreased concentrations or altered affinity for 25(OH)D, resulting in a higher proportion of free 25(OH)D. The effects of NB-UVB on serum and tissue levels of DBP in psoriasis patients should be investigated in future studies.

The baseline mean percentage of free 25(OH)D was 0.015%, rising to 0.017% after NB-UVB phototherapy. In healthy adults, percentages of free 25(OH)D from 0.02% to 0.09% have been reported [5,25]. Our results imply that psoriasis patients may have a lower percentage of free 25(OH)D compared to healthy individuals, but further studies are needed to confirm this.

We found no correlation between psoriasis severity and any of the vitamin D metabolites which were measured (free and total 25(OH)D and 1,25(OH)_2_D) at baseline or after NB-UVB). Thus, the hypothesis that free 25(OH)D correlates better with disease severity than total 25(OH)D could not be confirmed in this small cohort. However, it is important to note that the correlation between changes in 25(OH)D levels and psoriasis improvement is still not clear, but the evidence implies that NB-UVB therapy can provide multiple benefits for psoriasis patients.

Free and total serum 25(OH)D levels were positively correlated at baseline and after NB-UVB. This suggests that the accepted marker for vitamin D status, total serum 25(OH)D, is relevant for assessing vitamin D status in individuals with psoriasis. However, there was an increased dispersion of corresponding values for free and total 25(OH)D after NB-UVB compared to baseline. This could indicate an alteration in the equilibrium between free and total 25(OH)D as a result of NB-UVB phototherapy.

In line with previous results by Osmancevic et al., serum iPTH decreased significantly after phototherapy [22]. It has been suggested that iPTH is a better marker for vitamin D status than serum levels of total 25(OH)D. In this cohort, 30% of patients had sufficient levels of 25(OH)D, 40% had insufficient levels, and 30% had vitamin D deficiency at baseline [19], while all patients had iPTH levels within the normal range. Our findings illustrate the intricate and responsive regulation of iPTH orchestrated by changes in serum levels of vitamin D.

An inverse correlation between the percentage of free 25(OH)D and total 25(OH)D has been reported before and suggests a compensating mechanism altering the relationship between free and total 25(OH)D when needed [5,20,26].

Decreased blood pressure as a result of UVB exposure was expected and has also been shown before [27]. Hypertension and serum 25(OH)D levels have been reported to be inversely associated [28].

The main limitation of this study is the small number and the heterogenicity (regarding disease severity) of participants. Twenty patients were included of which 15 completed the entire protocol. Hence, the results must be interpreted with caution. The method used for measuring 25(OH)D levels (ECLIA) is not the gold standard method, such as liquid chromatography-tandem mass spectrometry (LC-MS/MS), but it is still a validated method and widely used in the assessment of vitamin D status [29]. To evaluate the true effect of NB-UVB it would have been optimal to exclude concomitant treatment with topical products, however, for ethical reasons, the patients were allowed to use emollients and mild topical steroids as a part of standard procedure. Six (30%) patients had sufficient levels of 25(OH)D at baseline which could limit the potential for a further increase by NB-UVB. However, a sub-analysis of these individuals’ 25(OH)D levels showed an increase of 50–83%.

## 5. Conclusions

NB-UVB phototherapy increases serum levels of total 25(OH)D, free 25(OH)D, and 1,25(OH)_2_D in psoriasis patients and is a clinically effective treatment. The effect on vitamin D metabolites is particularly relevant for psoriasis, as vitamin D might play a role in the pathogenesis. The increase of 25(OH)D levels after NB-UVB in this cohort was comparable to previous reports. The increase of total and free 25(OH)D remained significant in sub-analyses when stratifying for confounding factors for vitamin D status. We found no correlation between disease severity and vitamin D levels in this small cohort. The percentage of free 25(OH)D increased after NB-UVB implying that 25(OH)D not only increases but also becomes more bioavailable. This might be unique for UVB-induced vitamin D skin synthesis suggesting that NB-UVB therapy can provide multiple benefits for psoriasis patients. Total 25(OH)D seems to be a relevant marker for vitamin D status in psoriasis patients, but larger studies are needed to test the free hormone hypothesis in this group.

## Figures and Tables

**Table 1:** Demographic data including concomitant medication and possible confounders for vitamin D status at baseline for participating psoriasis patients.

	Mean	Min–Max	*n*
Age (years)	44.3	22.1–69.7	20
Age per sex women (years)	37.6	24.9–65.3	8
Age per sex men (years)	48.8	22.1–69.7	12
Weight (kg)	75.7	49.3–111	20
Height (cm)	172	154–189	20
BMI (kg/m^2^)	25.4	19.3–33.5	20
Systolic blood pressure (mmHg)	126	90–165	20
Diastolic blood pressure (mmHg)	79	60–95	20
Duration of psoriasis (years)	20	1–46	20
Hours spent outdoors per day during summer (winter)	5.6 (2.0)	1.5–12 (0.0–8.0)	18
Fish meals/week	1.5	0–3	18
	*n* (%)		
Current smokers	9 (45%)		
Omega 3 use	2 (10%)		
Obesity (BMI > 30 kg/m^2^)	3 (15%)		
Skin type			
II	8 (40%)		
III	12 (60%)		
Concomitant medication			
Antilipidemic use	1 (5.0%)		
Antihypertensive use	3 (15%)		
Antidiabetic use	1 (5.0%)		
Antidepressant use	2 (10%)		
Painkiller use	2 (10%)		
Thyroid hormone use	2 (10%)		
Hormonal contraception	1 (5.0%)		

**Table 2:** Serum levels of total 25(OH)D, free 25(OH)D, 1,25(OH)_2_D, and percentage of free 25(OH)D before (visit 1 = week 0) and after (when treatment was ended at weeks 10–12 = visit 3) narrow-band ultraviolet light B (NB-UVB) phototherapy in psoriasis patients. Due to five dropouts, the data is available for fifteen of the twenty included patients. Mean values ± standard deviation (SD) and *p*-values for the trend in time are presented.

	Before NB-UVB (*n* = 20)	After NB-UVB (*n* = 15)	Increase	*p*-Value
Total 25(OH)D (nmol/L)	63 ± 20	123 ± 23	95%	0.0002
Free 25(OH)D (pmol/L)	9.1 ± 2.0	20 ± 4.9	124%	0.0002
1,25(OH)_2_D (pmol/L)	87 ± 49	98 ± 29	13%	0.007
Percentage of free 25(OH)D (%)	0.015 ± 0.0031	0.017 ± 0.0029	9.2%	0.013

**Table 3:** Sub-analysis of total and free 25(OH)D serum levels before and after narrow-band ultraviolet light B (NB-UVB) phototherapy in psoriasis patients, stratified for confounding variables. Mean values ± standard deviation (SD) and *p*-values for the trend in time are presented. The number of patients (*n*) is presented as before (after) NB-UVB. Due to five dropouts, the data is available for fifteen of the twenty included patients. Before NB-UVB corresponds to visit 1 (week 0). After NB-UVB corresponds to when treatment was ended at visit 3 (weeks 10–12). BMI = Body Mass Index. PASI = Psoriasis Area and Severity Index. VAS = Visual Analogue Scale.

Variable	*n*	Total 25(OH)D (nmol/L)		*p*-Value	Free 25(OH)D (pmol/L)		*p*-Value
		Before NB-UVB	After NB-UVB		Before NB-UVB	After NB-UVB	
Gender							
Man	12(9)	64 ± 24	130 ± 25	0.008	9.3 ± 2.3	22 ± 4.5	0.008
Woman	8(6)	63 ± 14	113 ± 15	0.028	8.8 ± 1.5	18 ± 4.1	0.028
Age							
≤40 years	8(5)	52 ± 16	107 ± 9.3	0.043	7.9 ± 1.5	18 ± 3.1	0.043
>40 years	12(10)	71 ± 19	131 ± 24	0.005	9.9 ± 2.0	21 ± 5.4	0.005
BMI							
<30 kg/m^2^	17(13)	62 ± 21	123 ± 24	0.0015	9.1 ± 2.1	21 ± 5.0	0.001
≥30 kg/m^2^	3(2)	72 ± 13	126 ± 21	0.18	9.0 ± 1.6	18 ± 2.3	0.18
PASI							
<10	12(9)	62 ± 17	113 ± 19	0.008	8.8 ± 1.6	19 ± 5.3	0.008
≥10	8(6)	65 ± 25	138 ± 20	0.028	9.5 ± 2.5	23 ± 3.1	0.028
VAS							
≤7	6(5)	56 ± 28	119 ± 19	0.043	8.2 ± 2.2	19 ± 3.4	0.043
>7	14(10)	67 ± 16	125 ± 25	0.005	9.5 ± 1.9	21 ± 5.4	0.005
Smoking							
Yes	9(8)	55 ± 23	119 ± 25	0.012	8.5 ± 2.4	20 ± 5.0	0.012
No	11(7)	70 ± 16	127 ± 21	0.018	9.6 ± 1.6	21 ± 5.0	0.018
Disease duration							
≤20 years	10(6)	58 ± 18	109 ± 8.9	0.028	8.2 ± 1.5	17 ± 3.4	0.028
>20 years	10(9)	69 ± 21	133 ± 24	0.008	10 ± 2.2	22 ± 4.8	0.008

## Data Availability

The data that support the findings of this study are available from the corresponding author upon reasonable request.
